# Fundamental and experimental aspects of diffraction for characterizing dislocations by electron channeling contrast imaging in scanning electron microscope

**DOI:** 10.1038/s41598-017-09756-3

**Published:** 2017-08-29

**Authors:** H. Kriaa, A. Guitton, N. Maloufi

**Affiliations:** Laboratoire d’Étude des Microstructures et de Mécanique des Matériaux (LEM3), UMR CNRS 7239, University of Lorraine, 57045 Metz Cedex 01, France

## Abstract

Nowadays Field Emission Gun-Scanning Electron Microscopes provide detailed crystallographic information with high spatial and angular resolutions, and allow direct observation of crystalline defects, such as dislocations, through an attractive technique called Electron Channeling Contrast Imaging (ECCI). Dislocations play a crucial role in the properties of materials and ECCI has naturally emerged as an adapted tool for characterizing defects in bulk specimen. Nevertheless, fine control of the channeling conditions is absolutely required to get strong dislocation contrast for achieving comprehensive analysis. In this work, experiment-assisted fundamental aspects of the origin of dislocation contrast are studied. Experimentally, the potential of ECCI is explored in several dislocation configurations in Interstitial-Free steel (Fe − 1% Si) used as a model material. Full interpretations of dislocation contrast in (**g**, −**g**) and its evolution along the Kikuchi band are shown. Furthermore, a dislocation dipole is observed and fully characterized for the first time in an SEM.

## Introduction

After the discovery of the channeling phenomenon by Coates in 1967^1^, where the term “channeling” describes the electrons propagation trough the crystal to a higher depth before scattering, Booker *et al*. suggested that it could be possible to use this phenomenon for imaging defects under the surface of a bulk sample^2^. Therefore, an attractive technique called Electron Channeling Contrast Imaging (ECCI) was developed^3, 4^. It provides microstructure analyses on bulk samples where crystalline defects can be imaged with a visibility depth of about a hundred nanometers below the surface (the same order of magnitude of the thickness of a Transmission Electron Microscope –TEM – thin foil)^5^.

Although the contribution of TEM in materials science is incontestable^6^, its requirements have slowed its large-scale exploitation. These constraints have encouraged the adoption of techniques utilizing an SEM, an easier to use electron microscope. For instance, ECCI allows observation of crystalline defects over larger areas and offers the ability to execute diffraction contrast imaging inside SEM with sufficient imaging resolution to analyze individual dislocations (Burgers vector **b** analysis…) in bulk specimens generally without special preparation. This ability is due to the fact that modern Field Emission Gun (FEG-SEM) microscopes offer high imaging performance^7^ due to adapted characteristics such as high beam current mode, small beam convergence (quasi-parallel beam), and very small spot size that lead to high lateral resolution (a few nanometers) with a good signal-to-noise ratio (experimental details are presented in Methods section). Furthermore, accurate control of channeling conditions allows TEM style contrast analysis where the extinction criteria – **g**·**b** = 0 and (**g**·**b**) × **u** = 0 with **g** the diffraction vector, **b** the Burgers vector and **u** the dislocation line direction – are still applicable^8^.

Generally, literature reports that ECC images are obtained when the incident beam is oriented near a Kikuchi band edge where the BackScattered Electron (BSE) intensity is minimal. This corresponds to a channeling condition^3^. It has been shown that contrast of crystalline defects, such as dislocations, changes with the beam orientation near the band edge^9, 10^: when the incident beam is exactly oriented on the band edge, dislocations have a white/black contrast^4^. Conventionally, in this condition **s** ≈ 0, where **s** the deviation parameter, defines the deviation of the primary electron beam from the exact Bragg’s position. For **s** < 0, the beam is within the Kikuchi band and dislocations exhibit a weak contrast on a bright background. Contrarily, they appear very thin and bright on a dark background when the deviation **s** is slightly positive (**s** > 0)^10^.

Understanding the origin of the ECCI contrasts of defects and their evolution is therefore crucial. Only a few articles^4, 10^ report observations on the evolution of dislocation contrast by reversing the diffraction vector **g** (white side dislocations under +**g** diffraction become black under −**g** diffraction and vice versa). However, to our knowledge, none gives interpretations of these contrast modifications. Note that experimentally, the reversal of **g** is generally used for comprehensively analyzing dislocation dipoles in TEM^11, 12^.

In this paper, we report both fundamental and experimental aspects of diffraction for characterizing dislocations by ECCI in SEM. For illustrating our work IF steel is used because numerous dislocation analyses by ECCI have already been reported^9, 13^. Moreover, TEM literature shows that IFsteel contains dislocation dipoles as well^14, 15^.

Here we present ECCI analyses of IFsteel, slightly deformed in tension to 1%. The use of this technique combined with our contrast interpretations allows comprehensive analyses of dislocation contrast throughout the Kikuchi band and under the (**g**, −**g**) conditions.

## Dislocation contrast along a Kikuchi band

To understand contrast features in ECC images, one should define the role played by the term **g**·**b** in the contrast. Overall, a defect in the crystal produces a local displacement field noted **R**. This distortion of the lattice causes a phase factor exp (-iα) in the amplitude of the diffracted beam, where α = 2π**g**·**R**. The displacement field for a pure screw dislocation, for example, can be written as^16^:

1 $${\bf{R}}=\frac{{\bf{b}}}{2{\rm{\pi }}}{\tan }^{-{\rm{1}}}(\frac{z}{x})$$

Where z and x are the dislocation coordinates in the sample reference. It is noted from equation 1 that **g**·**R** is proportional to **g**·**b**.

On the Kikuchi diagram, bands are not isolated from each other. Effects of their contribution on the dislocation contrast have not yet been studied neither experimentally nor theoretically. In this part, this insight is developed. Here dislocations are observed with **s** positive (**s** small and **s** > 0 lead to thinnest dislocation image, see later).

**Figure 1:**
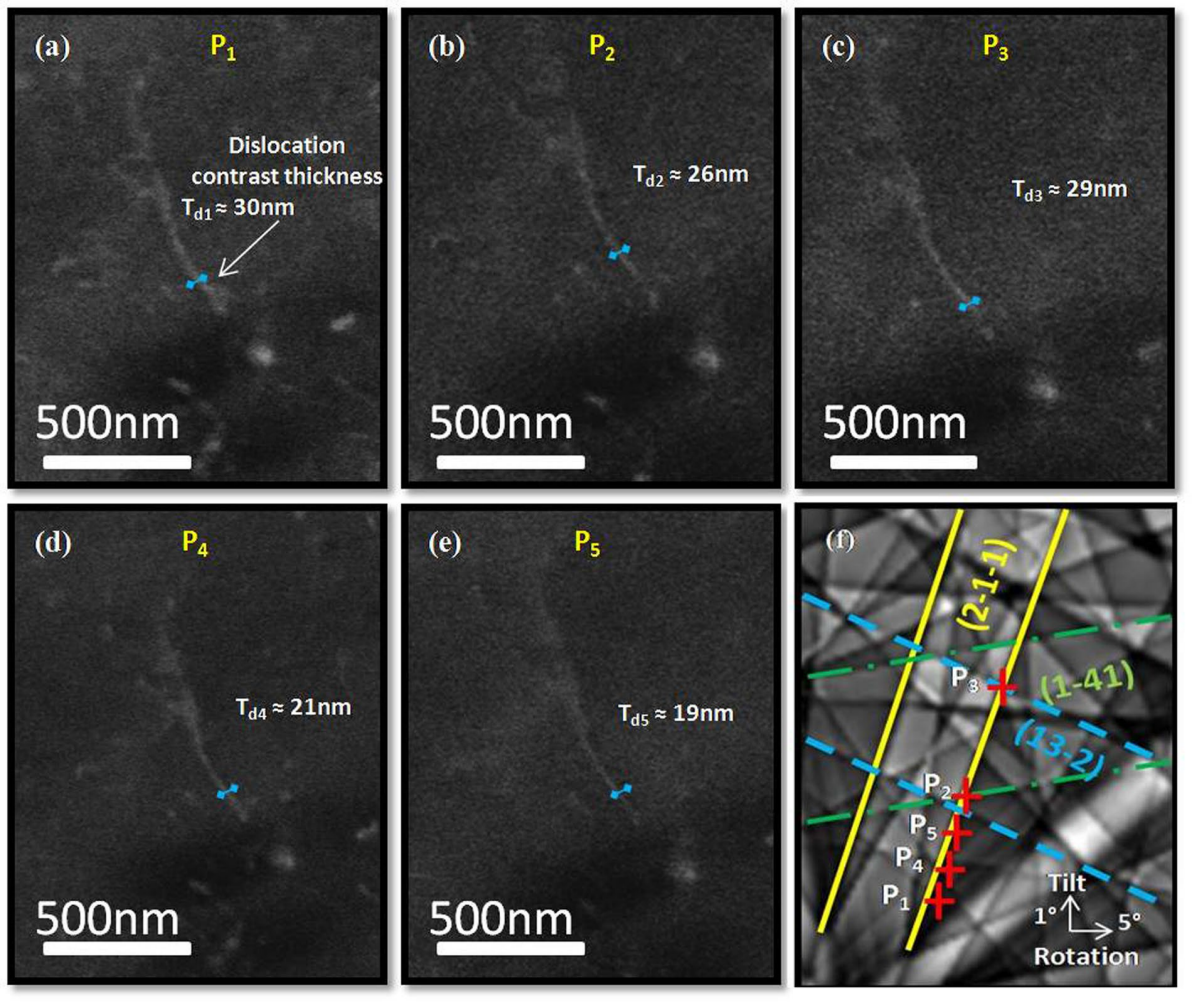
(**a**–**e**) ECC micrographs of single dislocation obtained with **g** = ($2\bar{1}\bar{1}$). (**f**) The simulated Kikuchi pattern presents the different incident beam positions, labeled P_i_. The colored straight lines present the main band (($2\bar{1}\bar{1}$) yellow) and two other bands (($1\bar{4}1$) green and ($13\bar{2}$) blue).

Figure 1(a–e) exhibit a single dislocation ECC micrographs obtained with the unique diffraction condition **g** = ($2\bar{1}\bar{1}$). The different incident beam positions P_i_ are labeled along the ($2\bar{1}\bar{1}$) band of the simulated Kikuchi pattern (Fig. 1f). Intensity line profiles perpendicular to the projection of the dislocation line (presented in blue on the dislocation line on Fig. 1) are obtained with ImageJ^17^. These obtained intensity profiles are fitted with a Gaussian curve to get the Full-Width-at-Half-Maximum (FWHM). Then, for every position P_i_, the image dislocation width T_di_ is deduced from the FWHM.

For P_1_, P_2_ and P_3_, the dislocation is thick (T_d1_ ≈ 30 ± 4 nm, T_d2_ ≈ 26 ± 4 nm and T_d3_ ≈ 29 ± 4 nm) compared to the other images taken at P_4_ (T_d4_ ≈ 21 ± 4 nm) and P_5_ (T_d5_ ≈ 19 ± 4 nm). For these latter (P_4_ and P_5_) the dislocation has the thinnest appearance obtained on a dark background. In these cases, the incident beam is oriented in a dark area far from any edges band intersection. In P_1_, P_2_ and P_3_, the main band ($2\bar{1}\bar{1}$) intersects others bands: the green ($1\bar{4}1$) band in P_2_ and the blue ($13\bar{2}$) band in P_3_ (Fig. 1f). To simplify the figure; the intersection band in P_1_ is not presented. All these bands contribute to the total BSE intensity and then the obtained dislocation is not thin. This evolution of the dislocation thickness has already been observed and explained in TEM^16^ but has not been reported in ECCI to date. This observed phenomenon is probably due to the complex contribution of more than one Kikuchi band for a given crystal orientation (corresponding to the incident beam position on the Kikuchi pattern).

Experimentally in ECCI, the most intense bands are generally used supposing that other bands with lower intensity are neglectable.

## Dislocation contrast in +g/−g diffraction

For the ±**g** diffraction, it has been reported, that the dislocation contrast is reversed *i*.*e*. black/white inversion^4, 10^. Here, interpretations are proposed for better understanding of the origin of the dislocation contrast for different situations: **s** ≈ 0, ±**g** and **s** slightly positive. Note that for **s** < 0 interpretation is not envisaged here because in this condition dislocations are in weak contrast on a bright background.

The BSE yield Δη contributes to the dislocation contrast. ‘Δ’ indicates that only the part of the total BSE intensity due to orientation contrast is calculated, while the contributions of atomic number or surface inclination are not considered. To explain the variation of this BSE signal as a function of the incident angle θ, a model as that of Reimer^18^ is used:

2 $${\rm{\Delta }}{\rm{\eta }}=\frac{{{\rm{N}}{\rm{\sigma }}}_{{\rm{B}}}}{2{\rm{\pi }}}{\rm{\xi }}{^{\prime} }_{0}(-\frac{{\rm{w}}+\frac{{\rm{\xi }}{^{\prime} }_{0}}{{\rm{\xi }}{^{\prime} }_{{\rm{g}}}}}{{1+({\rm{w}})}^{2}-{(\frac{{\rm{\xi }}{^{\prime} }_{0}}{{\rm{\xi }}{^{\prime} }_{{\rm{g}}}})}^{2}}+\frac{{\rm{w}}}{{1+{\rm{w}}}^{2}+{[{(1+w}^{2})(\frac{{\rm{\xi }}{^{\prime} }_{0}}{{{\rm{\xi }}}_{{\rm{g}}}})]}^{2}})$$

N is the number of atoms per unit of volume, σ_B_ is the scattering cross section for backscattering through angles larger than 90°, ${\rm{\xi }}{^{\prime} }_{0}$ and $\,{\rm{\xi }}{^{\prime} }_{g}$ are the absorption lengths, w is the tilt parameter (w = s ${{\rm{\xi }}}_{g}$ = g Δ${{\rm{\theta }}{\rm{\xi }}}_{g}$), g is the norm of **g**, (Δθ = θ − θ_B_) is the difference between the incident angle θ on the planes (hkl) and θ_B_, ${{\rm{\xi }}}_{g}$ is the extinction distance and **s** the deviation parameter.

### Deviation parameter s ≈ 0

**Figure 2:**
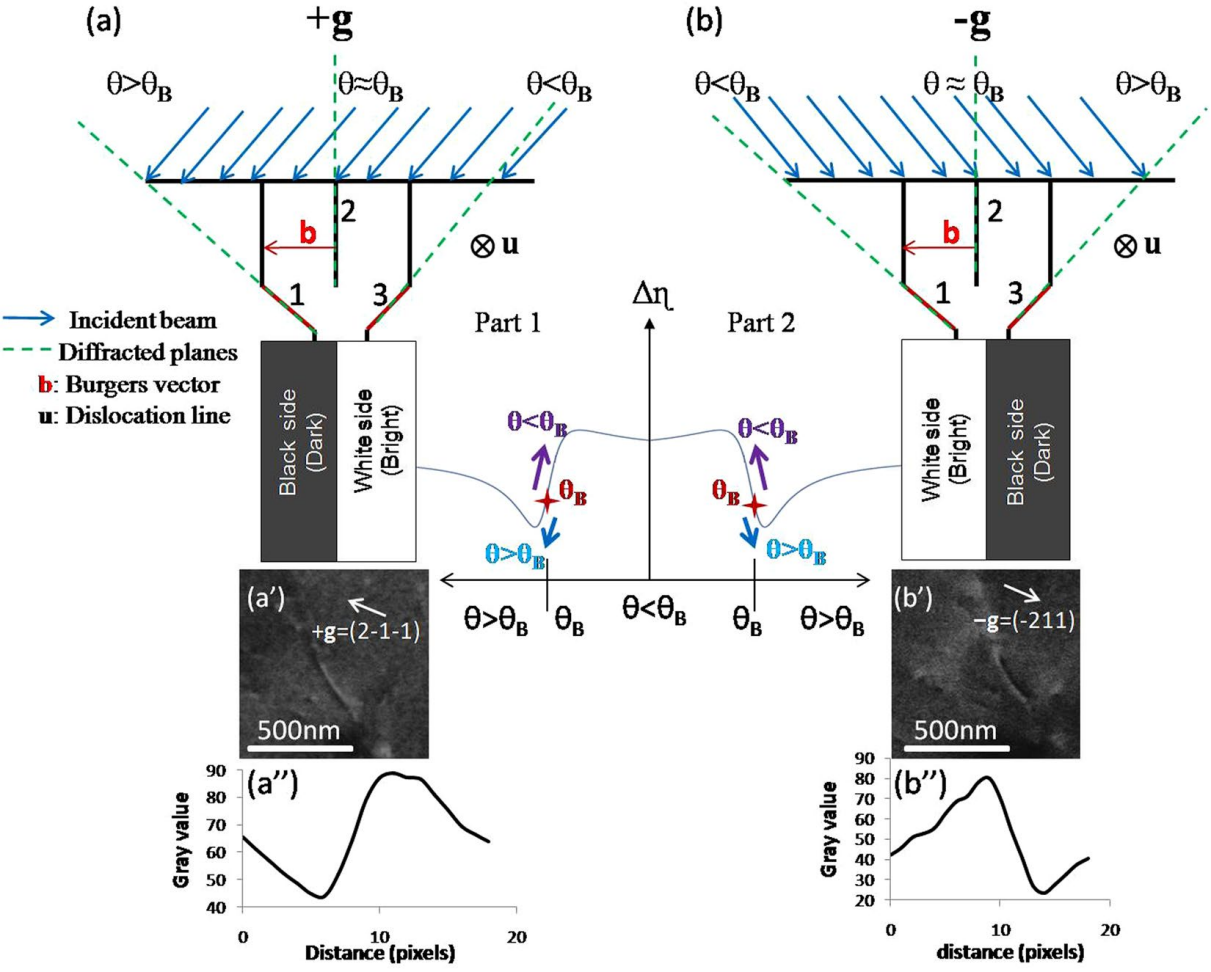
Schematic interpretations of diffraction (**a**) with +**g** and (**b**) −**g**. The BSE intensity modulation is presented by the curve for the two **g**. Note that the position of θ_B_ is obtained by equation 2: it corresponds neither to the hollow nor to the inflexion point of the curve. Dislocation contrast and their intensity profile are presented with (a’), (a”) +**g** = ($2\bar{1}\bar{1}$) and (**b**’), (**b**”) –**g** = ($\bar{2}11$).

When the incident beam is oriented at the edge of a band (**s** ≈ 0), a set of lattice planes are under the incidence angle θ equal to θ_B_. Bent planes around the defect are under incidence angles greater or less than θ_B_ (Fig. 2). This difference in θ influences the BSE yield and then the dislocation contrast.

Figure 2 is a schematic interpretation showing diffraction with ±**g** and their corresponding ECC micrographs. ECC images show the same dislocation obtained with **s **≈ 0 (**s** tends to 0 and θ tends to θ_B_). The curve in the center is obtained by plotting equation 2- substituting w by g Δ${{\rm{\theta }}{\rm{\xi }}}_{g}$- in function of θ (the Δη curve part 1 and part 2 correspond to the diffraction with +**g** and −**g** respectively).

For +**g**, the planes 1 and 3 are in incidence with θ > θ_B_ and θ < θ_B_ respectively. The plane 1 generates a low BSE signal (black side dislocation) compared to plane 3 (white side dislocation) because with θ > θ_B_, the BSE intensity tends towards decreasing values (blue arrow in curve part 1). Therefore, the dislocation contrast has black/white blocks. The bright part is on the same side as the plane 3 (Fig. 2a’ and a”). Inversely with −**g**, the planes 1 and 3 are in incidence with θ < θ_B_ and θ > θ_B_ respectively. Here, plane 1 generates a stronger BSE signal than plane 3 (purple arrow in curve part 2). The dislocation contrast is also presented as black/white blocks. The bright part is on the same side as the plane 1 (Fig. 2b’ and b”).

**Figure 3:**
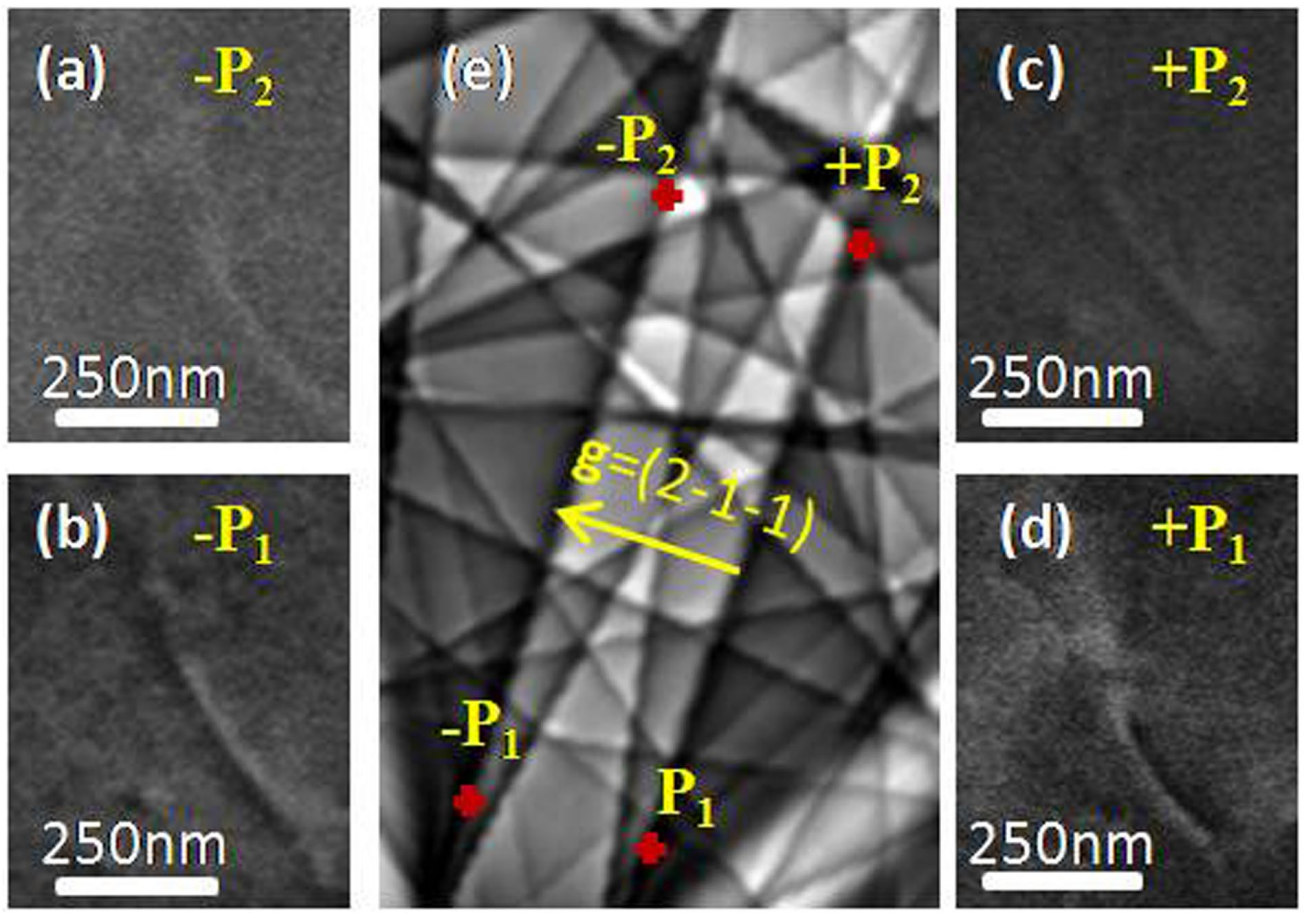
(**a**–**d**) ECC micrographs of the same dislocation observed with different position ±P_1_ and ±P_2_ presented in the simulated Kikuchi pattern in (**e**) for **g** = ($2\bar{1}\bar{1}$).

In this part, two positions +P_1_, +P_2_ and their opposites −P_1_, −P_2_ (localized on the opposite edge of the band) are presented on the simulated Kikuchi diagram (Fig. 3e). For each P_i_ micrographs of the same dislocation are recorded. For ±P_1_, the dislocation has a black/white sides and is reversed when the sign of **g** changes. On +P_2_, the dislocation also has a black/white contrast. Whereas on −P_2_, the incident beam orientation is within a lighter zone on the simulated Kikuchi pattern and the dislocation has a weak contrast on a bright background. Note that the position of the incident beam has an influence on the quality of the contrast as explained earlier (Fig. 1).

### Deviation parameter s > 0 (s is slightly greater than 0)

For getting the thinnest dislocation appearance with a high contrast on a black background, images are usually acquired with slightly positive deviation parameter **s** ^13^. In this case, θ = θ_c_ > θ_B_ (θ_c_ is the incident angle and the subscript “c” indicates channeling). A set of lattice planes are in incidence with θ = θ_c_ greater than θ_B_. Other sets are under incidence angles slightly higher or lower than θ_c_.

**Figure 4:**
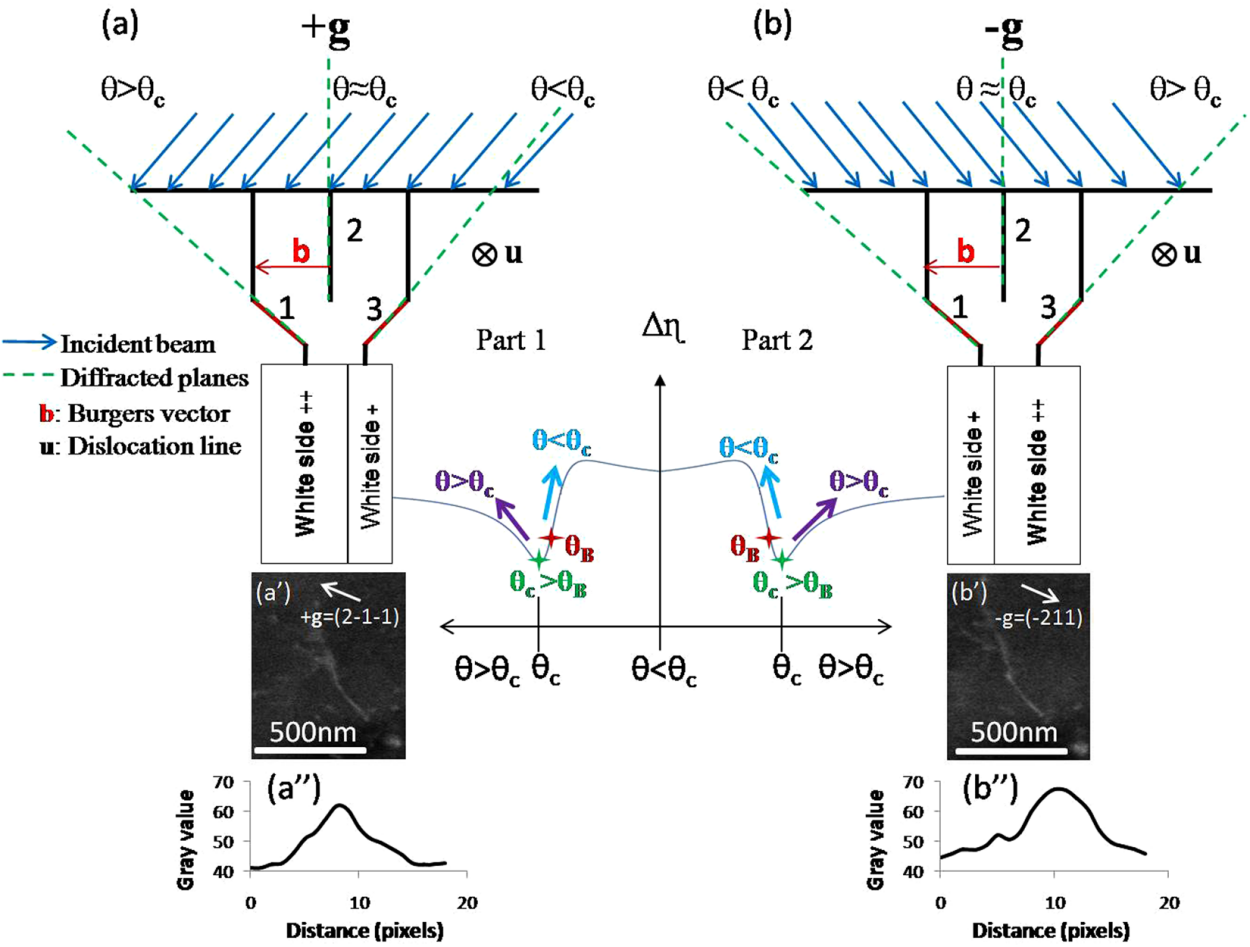
Schematic interpretations of diffraction in (**a**) +**g** = ($2\bar{1}\bar{1}$) and (**b**) –**g** = ($\bar{2}11$) with ECC micrographs of the same dislocation (a’), (b’) and their profile intensity (a”), (b”) respectively. The BSE intensity modulation is presented by the curve for ±**g**.

Figure 4a and b show schematic interpretations of diffraction with ±**g** and their corresponding ECC micrographs for the same dislocation. The planes 1 and 3 are in incidence with θ > θ_c_ and θ < θ_c_ respectively. Each of them generates an increasing BSE signal for both conditions ±**g** as it is presented by the curve of Δη as function of θ (arrows in the curves for the two parts). Therefore, the dislocation is bright for ±**g** (Fig. 4a’ and b’).

## Application for dislocation dipole characterization

**Figure 5:**
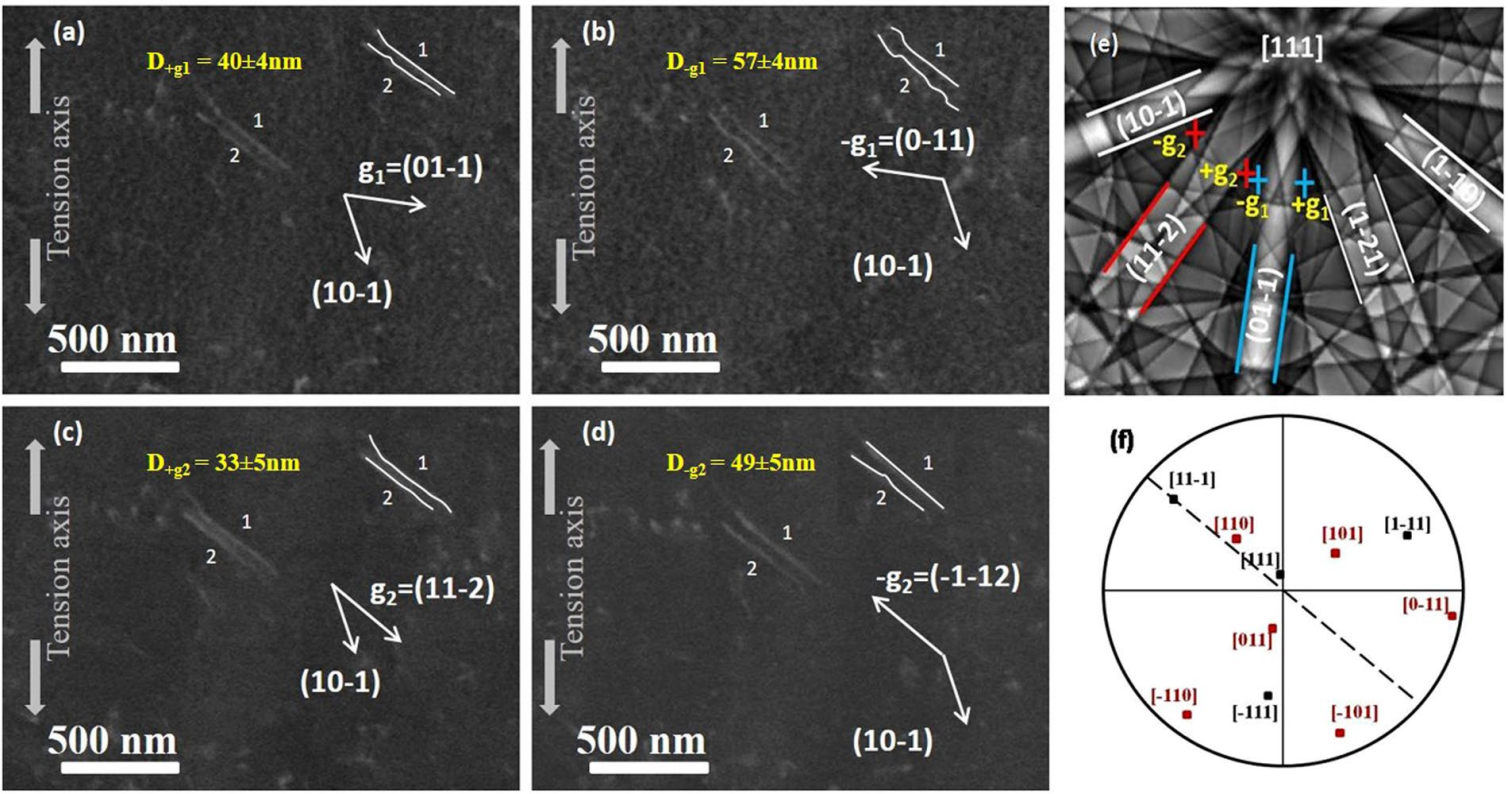
Micrographs of dislocations 1 and 2 observed with (**a**) **g**_**1**_ = ($01\bar{1}$), (**b**) −**g**_**1**_ = ($0\bar{1}1\,$), (c) **g**_**2**_ = ($11\bar{2}$) and (d) −**g**_**2**_ = ($\bar{1}\bar{1}2$) and their corresponding mean separation distances D ± **g**_**1**_ and D ± **g**_**2**_. This distance was deduced from several positions along the line, and considering a Gaussian dislocation profile. (**e**) Simulated Kikuchi pattern at 0° of the zone of interest and the different diffraction conditions ±**g**_**1**_ and ±**g**_**2**_ used in this study. (**f**) Stereographic projection showing the {111} and {110} poles, the dashed line corresponds to the projection of the dipole direction line.

ECCI observations are carried out with **s** slightly positive (**s** > 0). The region of interest is observed with different **g**: **g**_**1**_ = (${\rm{01}}\bar{1}$), **g**_**2**_ = (${\rm{11}}\bar{2}$), **g**_**3**_ = ($1\bar{1}0$), **g**_**4**_ = ($2\bar{1}\bar{1}$) and **g**_**5**_ = ($2\bar{1}1$) (Table 1). Figure 5 presents images of dislocation configuration observed with ±**g**_**1**_ and ±**g**_**2**_. These images show that two separated dislocations (1 and 2) are clearly identified. Three conditions must be satisfied for identifying a dislocation dipole:

1. Two single parallel segments of two different dislocations are clearly distinguishable. When tilting the sample one observes a finite distance between the both segments.
2. All dislocations segments are out of contrast with the same diffracting vectors. Therefore, the Burgers vector is unique for all segments and consistent with ±**b**.
3. Images obtained with +**g** and its opposite –**g** show that both contrasts are displaced in the opposite directions.

**Table 1:** Contrast of dislocation dipole under different diffraction conditions.

	${g}_{1}=({\rm{01}}\bar{1})$	${g}_{2}=({\rm{11}}\bar{2})$	${g}_{3}=(1\bar{1}0)$	${g}_{4}=(2\bar{1}\bar{1})$	${g}_{5}=(2\bar{1}1)$	Burgers vector **b**	Line **u**	α (°)
Dislocation 1	✓	✓	✗	✓	✗	$\pm \frac{1}{2}[{\rm{11}}\bar{1}]$	**[11** $\bar{{\bf{1}}}$ **]**	0
Dislocation 2	✓	✓	✗	✓	✗	$\pm \frac{1}{2}[{\rm{11}}\bar{1}]$	**[11** $\bar{{\bf{1}}}$ **]**	0

Note: ✓: visibility, ✗: invisibility; **b**: the Burgers vector, **u**: dislocation line, α: angle between **b** and **u**.

Both dislocations are out of contrast with **g**_**3**_ = ($1\bar{1}0$) and **g**_**5**_ = ($2\bar{1}1$). These extinction conditions are used to determine the Burgers vector, here ${\bf{b}}=\pm \frac{1}{2}[{\rm{11}}\bar{1}]$. Furthermore, the ECC micrographs obtained with different conditions ±**g**_**1**_ and ±**g**_**2**_ indicate that the reversal of **g** for both cases leads to a substantial change of the distance between these two dislocations images. The three conditions, cited above, are satisfied for these two dislocations and lead to the conclusion that the observed configuration is consistent with a dislocation dipole^11, 12^ (already observed by TEM in IF-steel) with ${{\boldsymbol{b}}}_{{\bf{1}}}=\pm \frac{1}{2}[11\bar{1}]$ and −**b**_**1**_ as Burgers vector for each dislocation respectively. The crystallographic line direction [$11\bar{1}$] of the obtained dipole is estimated from the trace line drawn as dashed line on the stereographic projection of the {111} and {110} poles. Therefore, the dipole is composed by screw dislocations lying in the ($\bar{1}10$).

**Figure 6:**
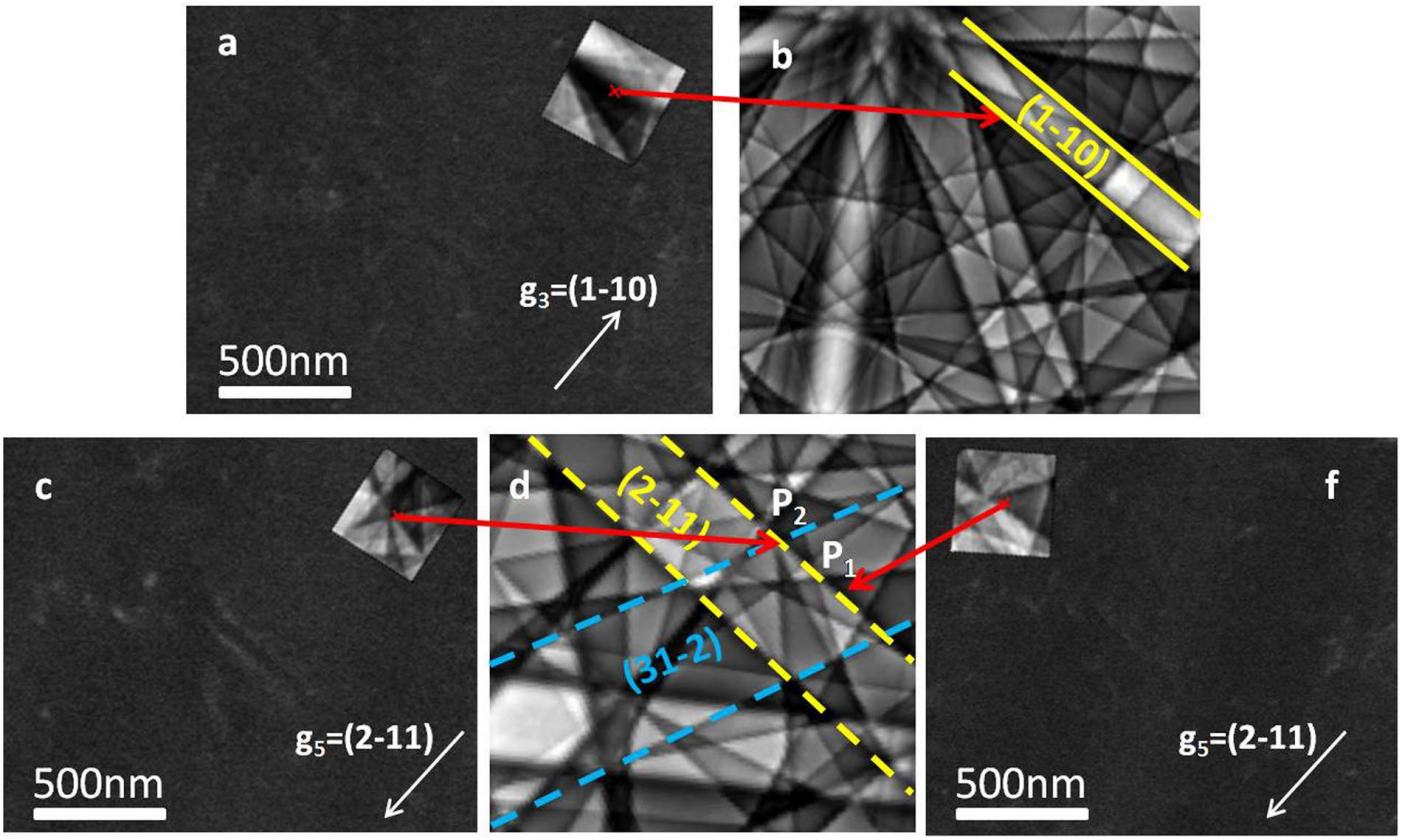
(**a**–**c**–**f**) ECC images for the dislocation dipole extinction with **g**_**3**_ and **g**_**5**_ (**b**–**d**). For each **g**, Selected Area Channeling Pattern (SACP) shows the incident beam orientation on the simulated Kikuchi pattern.

For **g**_**3**_ (Fig. 6a and b) the incident beam is oriented on the edge of the ($1\bar{1}0\,$) band. Dislocations are invisible. With ${{\bf{g}}}_{{\bf{5}}}=(2\bar{1}1)$, images are taken in two different positions P_1_ and P_2_ (Fig. 6d). Concerning P_1_, the incident beam is oriented on a dark area of the pattern. Dislocations are invisible (Fig. 6f). While in P_2_, the incident beam is oriented exactly at the intersection of (2$\bar{1}\,$1) and (31$\overline{2\,}$) band edges, the dislocations generate a contrast (Fig. 6c). In such a position, **g**_**5**_·**b** = 0 (extinction criterion) whereas for **g** = (31$\overline{2\,}$), **g**·**b** ≠ 0.

Analogous to TEM, the two-beam dynamical theory^19, 20^ tells us that the dislocation contrast is proportional to **g**·**b** ^16^. For P_2_ the dipole is clearly visible while the extinction condition **g**_**5**_·**b** = 0 is fulfilled. This misleading is explained by the fact that another band – where **g**·**b **≠ 0 – contributes to the contrast. Therefore, in the same manner as TEM, the two-beam condition is not satisfied.

To conclude, our fundamental studies bring new insights for complete analyses of dislocation contrast by ECCI. Nevertheless, resolving dislocations requires some conditions: (1) As the position on the Kikuchi band is very important, access to the orientation of the zone of interest with an accuracy better than 0.1° is mandatory; (2) the thinnest and the strongest dislocation contrast is obtained when the incident beam is oriented at the edge of an intense Kikuchi band with a deviation parameter **s** slightly greater than 0; (3) to obtain clear invisibility conditions, the incident beam should be oriented far from any Kikuchi band edge intersection.

Performing comprehensive dislocation analyses in an SEM without losing any information (compared to TEM) is naturally destined for a great future both in academic research (semiconductors^5^, metals^13^, rocks^21^) and in industry.

## Methods

The dislocation configurations are observed in IF steel slightly (1%) deformed in tension. After the deformation process, the specimen surface was ground to 4000 grit followed by mechanical polishing with 1 µm diamond paste. Finally, a mechano-chemical polishing was performed using 50 nm colloidal silica. Prior to the dislocation observation, a crystallographic orientation map is obtained by Electron Backscattered Diffraction (EBSD) in a Zeiss SUPRA 40 microscope with an accelerating voltage 20 kV. In EBSD, the sample is positioned at a working distance of 15 mm and tilted to 70°. The orientation of the grain of interest is presented by the Euler angles. These latter are used to simulate an EBSD pattern on the “Esprit DynamicS” software from Bruker. The pattern is simulated at 0° because in ECCI our specimen is placed at 0°.

Obtaining the crystallographic orientation of regions of interest is a preliminary step to ECCI that requires an accuracy for crystal orientation of 0.1° ^22^. To get this aim, Zaefferer *et al*.^9^ applied the EBSD. However, due to the particular geometry of EBSD and to inaccuracies of stage tilt, the obtained EBSD-Kikuchi pattern offers an absolute angular accuracy of 1–2° approximately^23^. For this purpose, H. Mansour *et al*.^13^ proposed an innovative procedure using “High Resolution Selected Area Channeling Pattern (HR-SACP) for precisely controlling the channeling conditions. The same team therefore developed the rocking beam mode on the GEMINI column of Zeiss SEM allowing the collection of SACP of an angular range 4.4°, an accuracy for the orientation better than 0.1° and for the first time locally reaching high spatial resolution (≈500 nm)^24^. The HR-SACP is superimposed on the EBSD pattern in order to determine accurately the orientation. Finally, to satisfy the different diffraction conditions, the sample placed on the microscope stage is tilted (for example up to 21° for given conditions) and rotated. Dislocations imaging by ECC is carried out for 5 minutes using a four quadrant Si-diode backscattered electron detector, an acceleration voltage of 20 kV, a beam current of 80 µA (with a probe current ≈736 pA) and a working distance of 7 mm.
